# Momentum-locked spin between topological and defect states in 1D patterns on bilayer graphene

**DOI:** 10.1038/s41598-025-12215-z

**Published:** 2025-08-01

**Authors:** R. Guerrero-Avilés, A. Ayuela, Leonor Chico, W. Jaskólski, M. Pelc

**Affiliations:** 1 Centro de Física de Materiales-Material Physics Center CFM-MPC, Donostia International Physics Center (DIPC), Paseo Manuel Lardizabal 4-5, 20018 Donostia-San Sebastián, Spain; 2 TECNALIA, Basque Research and Technology Alliance (BRTA), 48160 Derio, Spain; 3 GISC, Departamento de Física de Materiales, Facultad de Ciencias Físicas, Universidad Complutense de Madrid, 28040 Madrid, Spain; 4 Institute of Physics, Nicolaus Copernicus University in Toruń, Grudziadzka 5, 87-100 Toruń, Poland

**Keywords:** Condensed-matter physics, Electronic properties and materials, Topological matter, Graphene, Nanoscale materials

## Abstract

Gating Bernal bilayer graphene breaks the inversion symmetry so that the stacking AB/BA boundaries within the gap reveal topologically protected states. In this study, we theoretically investigate arrays where the AB and BA domains are periodically patterned with experimentally identified defect lines. In the calculations we consider electron-electron interaction effects using density functional theory. Our findings reveal the existence of topological states within a gap induced by the patterning without an applied gate voltage. Furthermore, with an applied gate potential, the defect lines introduce spin-polarized states pinned within the gap and exhibit ferromagnetically coupled states. Importantly, we observe a hybridization of magnetic and topological states near the valleys that form conducting channels characterized by spin-momentum locking. The effect persists even with slight n-doping and gate voltage; however, the progressively pinned n-doped defect states induce spin polarization in the topological and valley states. Additionally, the two-dimensional bands under doping conditions exhibit nesting across the Fermi surface, allowing for modulation of charge densities along the lines which are nearly commensurate with the underlying graphene-defect lines. These quasi-one-dimensional patterns in bilayer graphene show a new kind of spin-conducting channels with novel characteristics common to both spintronics and valleytronics.

## Introduction

Graphene electronic properties can be finely tuned through its interaction with other layered materials, including graphene itself^1,2^. The number of layers and their relative stacking determine a diverse range of electronic and optical properties. In its most stable form, known as Bernal AB stacking, bilayer graphene (BLG) exhibits a parabolic band structure close to the Fermi energy, and this band structure features a tuneable gap with gates. The properties of the gapped bilayer are notably changed by introducing a domain wall that separates the AB stacking from its mirror reflection BA. Along the boundary of this domain wall, efficient conducting channels emerge. In fact, the application of gate voltage opens a gap in which two gapless states for each valley appear, being topologically protected^3–7^. The transition between the two distinct stackings is performed by different means such as corrugations^8–10^ and the application of shear and tensile strains^11,12^, methods that smoothly connect the graphene lattice on both sides of the domain wall. An alternative approach involves the creation of large AB and BA domains in bilayer graphene through controlled gating^13^ and twisting^14,15^. Another way to join the mirror Bernal stackings is to employ atomically sharp lines of defects within a layer, such as grain boundaries of pentagons and octagons (8-55)^16,17^. These types of boundaries break the local sublattice symmetry, concurrently introducing the so-called defect-localized states. Consequently, assembling graphene structures with grain boundaries to fabricate bilayers produces a complex interplay between states arising from the grain boundaries and those typically originating from the stacking change with topological character.

Quasi-one-dimensional defect lines in graphene have emerged as intriguing candidates for potential materials in future nanoelectronics^18,19^. These defect lines were observed in monolayer graphene^16^ and subsequently investigated using density functional theory (DFT) and tight binding models, which incorporate on-site Coulomb repulsion^20^. Remarkably the defect bands can be linked to the edge states found in nanoribbons with zigzag edges, which have been studied extensively; these defect bands generally originate in the defect-related interface between graphene grains^21^. These one-dimensional systems due to the interfaces show localized states near the Fermi level that have significant electronic and magnetic properties. For instance, in the context of graphene ribbons, these localized states have interesting transport and magnetic properties when considering electron-electron interactions^22,23^. Therefore, it becomes imperative to recognize that the states introduced by defect lines in bilayer graphene are compelled to interact with the topological states induced by the stacking changes described above. Furthermore, a comprehensive understanding of this intricate interplay requires including electron-electron interactions to precisely describe this complex electronic behavior.

In this work, we calculate the electronic structure and explore the interaction between defect and topological states in an array of pentagon-octagon defect lines in bilayer graphene. We begin by establishing a fundamental model for the defect states in conjunction with the topological valley states, which is then compared with results obtained using the tight-binding approximation. Furthermore, our comprehensive analysis of electronic, magnetic, and response properties studied using the DFT approach unveils intriguing physical phenomena, and shed light on the pivotal role of electron-electron interactions. Notably, we identify a unique point where spin bands intersect, where their momenta are locked owing to their spin polarization. Additionally, we consider the behavior of the bands under the influence of electric fields and n-doping, which enables us to analyze the further interactions between the topological and defect-related states in engineering applications.

**Figure 1:**
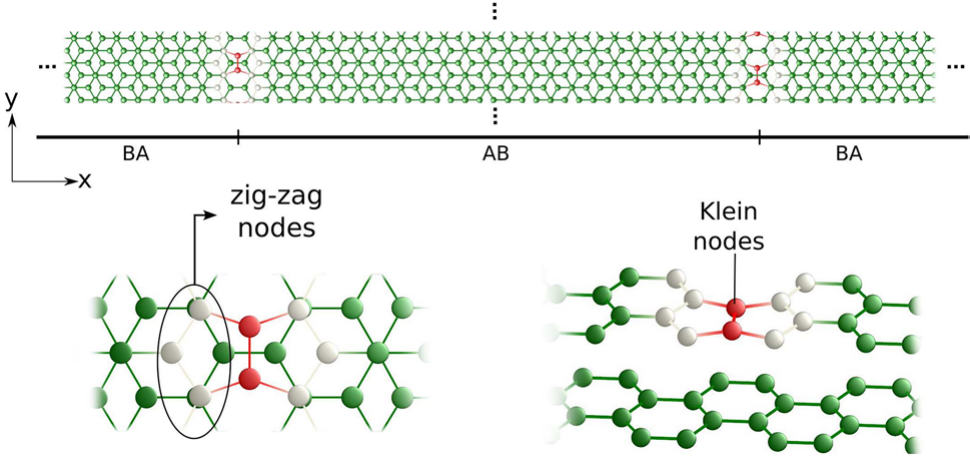
(Upper panel) Schematic view of an array of defect lines in bilayer graphene separating AB and BA stacking domains. (Lower panels) Zoom views of defect nodes. Gray and red carbon atoms correspond to zigzag- and Klein-like nodes, respectively.

## Structure and basic theory models

### Structural model

We begin by describing the geometric arrangement of an array of defect lines in Bernal-stacked bilayer graphene, illustrated in Fig. 1. In this configuration, the supercell accommodates two stacking domain walls, each associated with a pair of topological defect lines. These defect lines alternate between an octagon and a pair of pentagons, forming grain boundaries that define the AB and BA-stacked regions within bilayer graphene. The defect states along the 5-5-8 grain boundary in monolayer graphene can be viewed as valley-protected modes arising from a Berry phase mismatch across mirror domains. However, since they connect the valley K to K’, their valley conservation is limited in comparison to the topological protection of gapless states in AB/BA stacking changes in bilayer graphene. To represent the array of bilayer graphenes, we employ a specific supercell with $L = 24$ unit cells along the *x*-direction, having the cell of graphene nanoribbons as the unit length. The defect line itself can be visualized as connecting two distinct graphene edges, namely, a pristine zigzag edge and a Klein-terminated edge^24^. Consequently, we label the defect nodes as zigzag and Klein, as illustrated in the lower panels of Fig. 1. Practically, every alternate pair of Klein nodes is dimerized and connected, a phenomenon that has been experimentally observed in a single layer of graphene^16^.

### Basic ingredients of a model

To gain insights into the hybridization between the defect and topological states, we begin by constructing a straightforward model. The effective Hamiltonian is expressed as:

1 $$\begin{aligned} \mathcal {H}(k)= \begin{bmatrix} \begin{array}{cccc|cc} k+V & 0 & \gamma _1 & 0 & 0 & 0 \\ 0 & -k+V & 0 & -\gamma _1 & \gamma _2 & 0 \\ \gamma _1 & 0 & k & 0 & 0 & \gamma _2 \\ 0 & -\gamma _1 & 0 & -k & 0 & 0 \\ \hline 0 & \gamma _2 & 0 & 0 & H(k)k & 0 \\ 0 & 0 & \gamma _2 & 0 & 0 & H(k)k \\ \end{array} \end{bmatrix} \end{aligned}$$

In this representation, the upper 4*x*4 block encapsulates the bilayer graphene Hamiltonian, tailored for the topological states. This Hamiltonian accounts for the two graphene Dirac cones within each layer, showing the diagonal $\pm k$ states. The layers interact through the coupling parameter $\gamma _1$, and denotes the applied gate voltage^25^. The $\gamma _1$ parameter enables the mixing of states with parallel momenta, with the same sign of *k*, thereby maintaining them within the gap induced by *V* (Note that applying a voltage *V* and mixing *+k* and* -k* states between layers leads to the opening of a gap in bilayer electron bands, showing the so-called “Mexican shape.”). The parameter $\gamma _2$ denotes the coupling between defect-localized states and opposite valley states (K and K’), distinct from the interlayer hopping $\gamma _1$. These states correspond to the description of topological states. The electronic dispersion for bilayer graphene is derived from the Hamiltonian presented in Eq. (1), with $\gamma _1$ set to 0.1 $\gamma _0$ eV to characterize the interlayer interaction being $\gamma _0$ the hopping parameter of monolayer graphene. The lower 2*x*2 block incorporates the defect states involving two diagonal terms, *H*(*k*)*k*, where *H*(*k*) is the Heaviside function. This function accounts for the dispersion near the K and K’ valleys, effectively intertwining through the $\gamma _2$ coupling parameter with $\pm k$ states which have opposite momentum and valley features in each layer. This model serves as a basic framework for our following analysis, and enables us to perform an initial study of the intricate interplay between defect and topological states in bilayer graphene.

**Figure 2:**
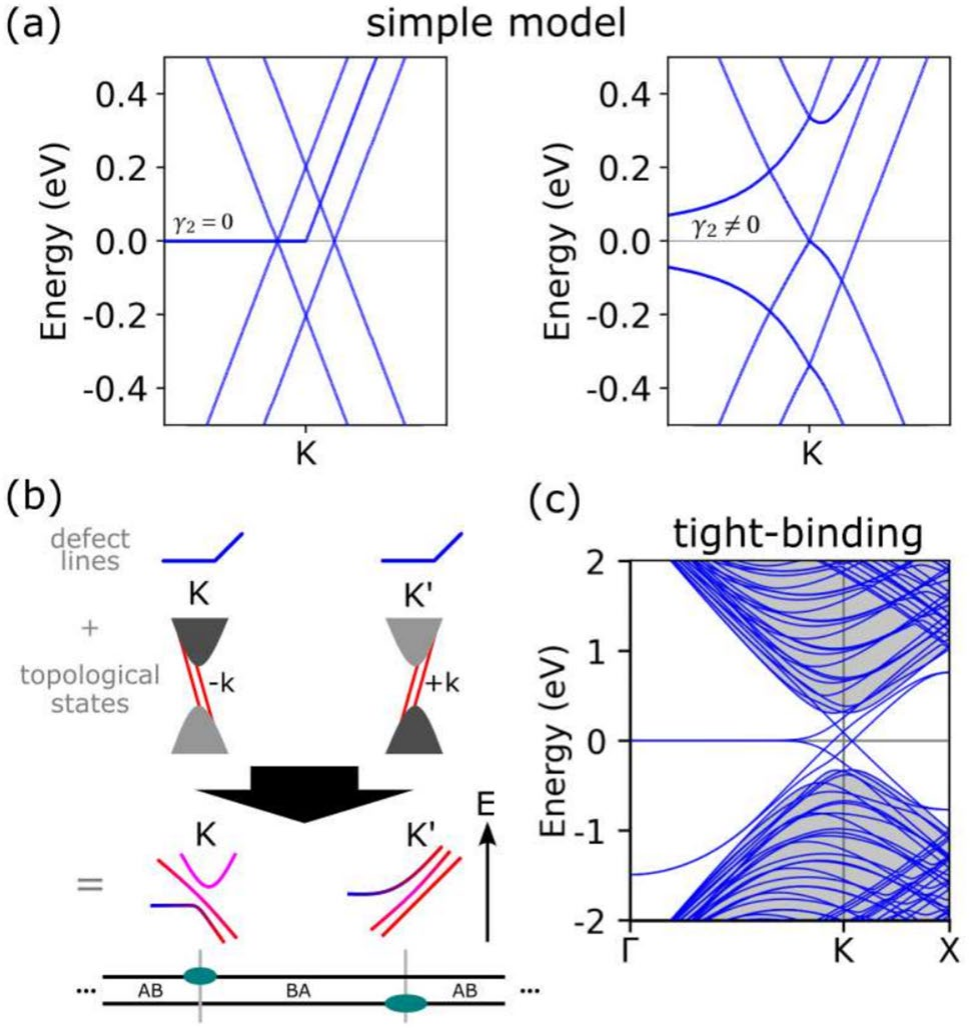
(**a**) Model bands for zero and non-zero $\gamma _2$ value. (**b**) Scheme of hybridizations of gapless states around both valleys. (**c**) Tight-binding band structure of the array of defect lines shown in the previous figure.

Figure 2a in the left panel displays the electronic dispersion obtained from the Hamiltonian in Eq. (1), where we set $\gamma _2$ to zero. The introduction of interlayer coupling ($\gamma _1$) leads to the topological gapless states, which are the hallmark of the change from AB to BA bilayer stacking^4,26^. These states bridge the valence and conduction bulk bilayer bands with two pairs of bands, each having opposite velocities which are associated with the K and K’ valleys. We note that when both Dirac cones have an energy gap due to the applied voltage *V*, the topological states are kept in the middle.

We extend our analysis to the scenario with a non-zero $\gamma _2$ value, given specifically as $\gamma _2 = 0.075 \gamma _0$ eV, and show the electronic dispersion in the right panel of Fig. 2a. In this configuration, where the defect states are coupled with the topological states, we observe that a pair of the previously mentioned topological states exhibits a $+k$ dispersion. The defect states symmetrically split away from the Fermi level and display opposite dispersion patterns. This behavior stems from their interaction with topological states characterized by different velocities associated with their distinct sign values of *k*.

The distinctive behavior exhibited by the defect bands can be attributed to the stacking changes that are inherently connected to two opposing valleys, that correspond locally to each stacking domain, as described in Fig. 2b. It is noteworthy that each stacking change in bilayer graphene essentially mirrors the other by changing its relative chirality. Since the defect lines reside in different layers, they become entangled with the opposite valleys, namely, K and $\text{K'}$. However, the interaction between defect lines, as being flat bands, requires to include electron-electron interactions because they play a crucial role in understanding the intricacies of the electronic structure around the bilayer valleys, a topic to be discussed below.

### Band structure within the tight-binding approach

After our development of the simple model elucidating the interaction between the defect and topological states, we present the electronic band structure of the investigated defect line array. We consider the atomistic structure through tight-binding calculations, as shown in Fig. 2c (The tight-binding electronic dispersion is calculated using the hopping factor between nearest carbon $\gamma _0$= − 2.66 eV and the intralayer hopping $\gamma _1$ = 0.1$\gamma _0$ eV.). The bulk bands at the K point show a characteristic parabolic curvature and are shadowed in gray; they have a gap stemming from the periodical stacking change induced by the defect lines. Within the gap we observe flat localized bands near the Fermi level along the $\Gamma$ path. Specifically, these states at the $\Gamma$ point are located along the defect lines. As they approach the K valley, these defect bands gain dispersion and hybridize with the valley states. Note that near the Fermi level the tight-binding bands bear a remarkably similar pattern to those obtained in the model. Thus the TB bands close to the K valley result from their hybridization with the topological states as explained above for the model.

## Results and discussion

### Momentum-locked spin-crossing

In this section, we present the results of our DFT simulations performed for the array shown in Fig. 1, employing the SIESTA method^27^ and carried out with a van der Waals functional^28,29^ already used previously^30,31^. Note that electron–electron interactions are treated within DFT via the exchange-correlation functional, as has been performed in similar systems^22,23,32–34^. The three-dimensional grid of density and exchange-correlation potentials uses a mesh cutoff of 600 Ry. To precisely describe the electron distribution near the Fermi level, especially at the $\Gamma$ point, we chose a minimal electron smearing of about 0.1 meV. The corresponding converged mesh grid in the reciprocal space becomes thus quite extensive, comprising *101x15* *k*-points to ensure convergence for the relaxed geometries. We performed simulations relaxing the input structures and computing their electronic properties. The relaxed geometry has in-plane carbon-carbon distances of about 1.44 Å, with slight variations near the defect line. The most deformed hexagons share edges with octagons and pentagons and undergo the most significant deformations with shared lengths of 1.48 and 1.41 Å, respectively. Further details regarding the relaxed structure, along with the corresponding C-C distances, can be found in the Supplementary Information (SI).

**Figure 3:**
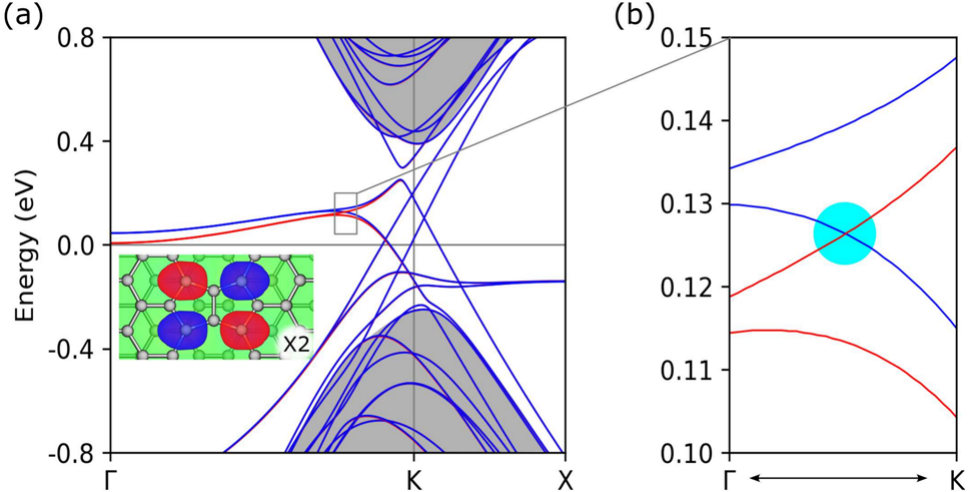
(**a**) Electronic band structure of the defect line array. The gray-shaded regions define the bilayer bands, which are characterized by their curvature, and exhibit a distinct energy gap. At the Fermi level, the nearly flat states display spin splitting. The inset shows a specific case of the wavefunction at the $\Gamma$ point, namely a spin-up state. This particular state is localized at a defect in the upper layer and it is degenerated with another state localized close to the second defect line in the bottom layer. It is noteworthy the presence of a spin-crossing point located near the K valley and marked by a cyan dot in the zoomed-in right panel (**b**).

We begin by examining the electronic band structure, and investigate the localization of defect states. Figure 3 shows the band structure of the defect line array. We observe a band gap opening between the bilayer bands even in the absence of external gating. The gap opening is characteristic of superlattices created through periodic distortions during the patterning of other materials onto graphene substrates. Furthermore, the array patterning introduces a long wavelength leading to an interplay between periodicities within the folded bands. These bilayer bands are thus divided into a series of subbands within the upper and lower part of the bilayer valleys, visible as several sets of blue bands included in the grey area, also shown in Fig. 2c. In fact, the band gap can be understood as a result of the array structure, which effectively behaves as a periodic assembly of stripes characterized by alternating positive and negative potentials.

Within the energy gap around the K point, we observe several gapless states nested among the bilayer bulk bands. Four intersecting bands are seen, organized into two pairs, with each pair related to one of the stacking changes. These four bands originate from the topologically protected states characteristic for the defectless stacking domain walls^8^. However, as we move away from the K point, these topological bands hybridize with the localized states due to the defect lines. The nature of this hybridization is intricately dependent on the details of the localization of the two types of states, as they interact primarily within the same layer and sublattice^35^.

The bilayer and topological states show spin degeneracy except for the two bands near the Fermi level, spanning from $\Gamma$ to *K*. These two bands define states localized mostly along the defect lines. Characterized by being nearly flat and partly occupied, their limited dispersion is strongly influenced by electron-electron interaction: these bands also remain fully flat and partly occupied in calculations within the tight-binding approximation, as depicted in Fig. 2c. Each of the defect bands is associated with the defect lines located in both side graphene layers. An example of the wave functions at the $\Gamma$ point for the upper layer is shown in the inset of Fig. 3a. These defect bands show strong localization at the zigzag nodes on both sides of a defect line. This localization distinguishes them from the edge states observed in zigzag-terminated nanoribbons, which are predominantly confined to a single edge^20,22^. These defect bands hybridize with a pair of topological states related to the stacking change near the valleys, provided that they share the same momentum.

The hybridized defect and stacking-change topological states show a spin-crossing point, marked in Fig. 3b with a cyan dot. The carrier momenta related to the two bands have opposite signs, which implies spin-momentum locking. This locking refers specifically to the crossing point near the K valley, where spin-up and spin-down states exhibit opposite velocities; other bands may share velocity signs and do not exhibit this locking. We note that the wave functions of the two crossing bands are localized in different layers. Thus, currents related to spin-up and spin-down channels flow in opposite directions and are also spatially separated in different graphene layers. Although defect and valley states overlap in energy, they can be distinguished experimentally via spin- and layer-resolved STM or transport probes. Their spatial localization in opposite layers and distinct spin textures allow for selective addressing, particularly under asymmetric gating. The layer-resolved spin transport makes this array a possible candidate for engineering based on the spatial layer separation of the spin currents^25^. Certainly, disorder and impurities in average might modify the characteristics of the 1D channels as shown in Ref.^36^, and should be considered in future studies, but in general they will not be substantial modifications of the effects described here.

### Parallel couplings of defect line spins under gates

**Figure 4:**
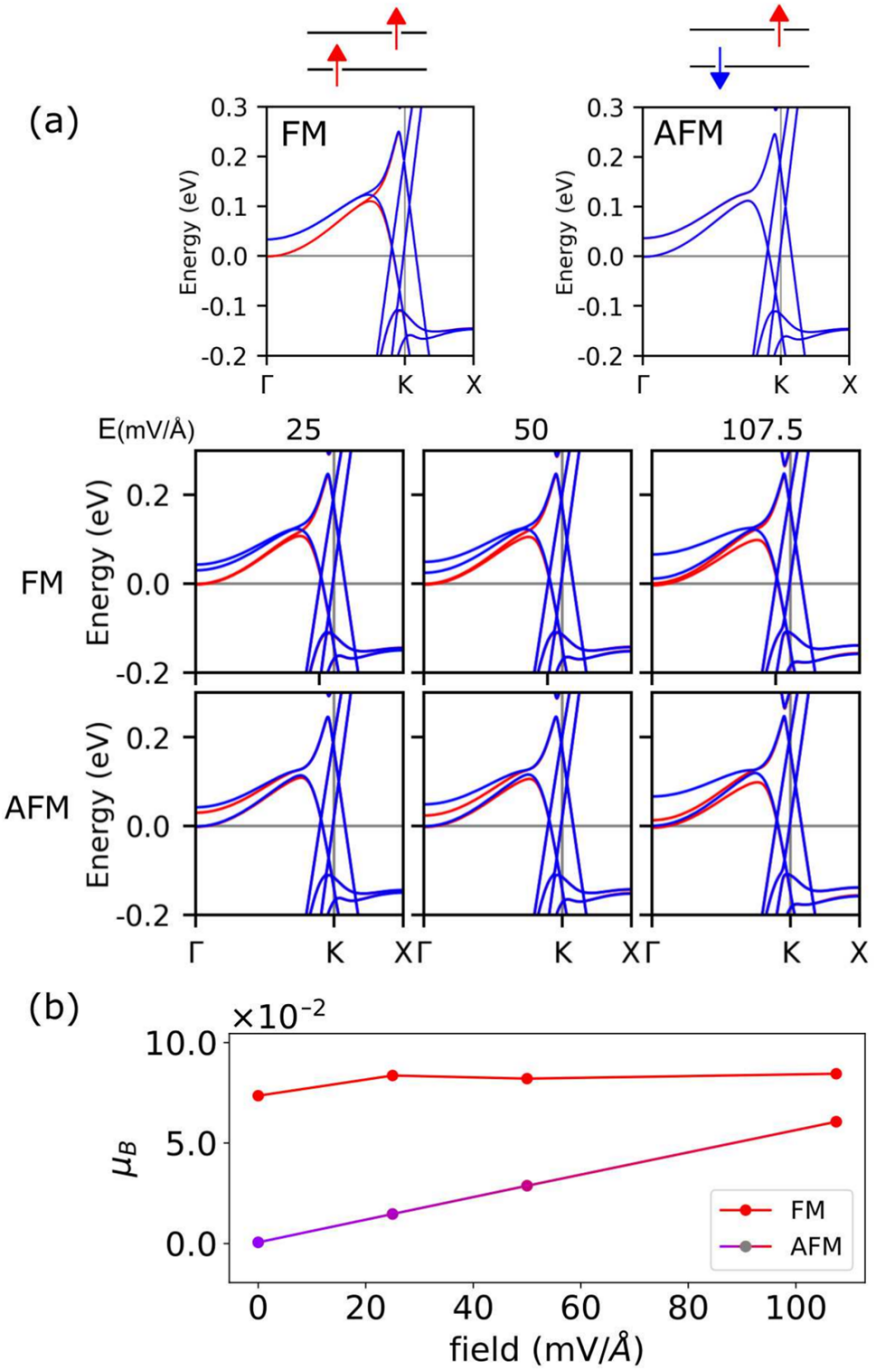
(**a**) Evolution of the band structure under an applied electric field for the FM and AFM magnetic couplings between the defect lines. In both cases the bands reach saturation for a value of the field $E\overset{>}{\sim }\ 100$mV/Å. (**b**) Total magnetization versus field for the initial FM and AFM couplings. Note that in the AFM case the color changes from blue to red because the spins become ferromagnetically coupled with applied gates.

The spin coupling of defect lines in the simulations above is slightly stabilized in the ferromagnetic (FM) configuration which has the lowest energy. We now proceed to perform FM-AFM calculations with varying fields, as shown in Fig. 4. The initial spin configurations establish FM and AFM couplings for the zigzag node lines as displayed in the upper schemes. The upper panels of Fig. 4a compare the band structures of the FM and AFM phases at zero field. The two configurations are nearly identical, differentiated by the spin-degeneracy in the AFM case along the $\Gamma -K$ path. The AFM case exhibits fully degenerate bands with each layer having spin polarization of opposite sign. The effect of an electric field on FM and AFM spin line couplings is then examined, with a discussion on the two distinct spin configurations under the electric field. We apply the field perpendicular to graphene layers with values of 25.0, 50.0, and 107.5 mV/A. Note that such inversion symmetry emerges in the periodic superlattice due to the alternating placement of defect lines in the upper and lower layers. This leads to equivalent physical responses under opposite-polarity electric fields, similar to those observed in arrays of alternating stacking domains.

**Figure 5:**
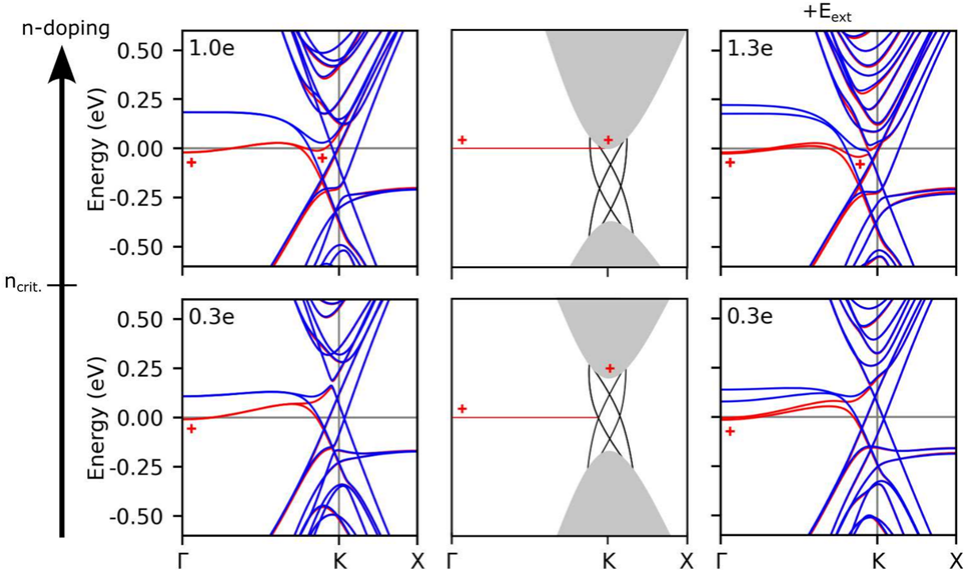
Band structure with doping values, *n*, ranging from 0.3e to 1.3e (left) without and (right) with an electric field $E = 150$ mV/Å. The schematic illustration in the middle panels depicts how n-doping not only fills the defect bands, but also is pinning them. Red and blue curves represent defect states and valley-related bands separated in up and down spins, respectively. The topological and valley states become occupied and spin-polarized, indicated with the + mark.

Figure 4a shows how the band structures for both magnetic phases change as the electric field is increased. The bulk bands remain almost spin-degenerate and the impact of the field effect can be mostly observed on the defect flat bands. In the FM case the field splits the two spin-down bands and shifts one of them down in energy. The spin-up bands remain pinned at the Fermi level with an almost constant spin polarization value. Band splitting also occurs for the AFM coupling in the defect bands, which are degenerate for spin-up and down polarizations at zero field. In this coupling scenario, a pair of bands splits and the one with spin-up polarization is shifted downwards in energy. In fact, the splittings increase and saturate for field $E$ values above 100 mV/Å, where the band structures for the FM and AFM couplings become similar. Although the order of the bands is different, the two middle bands nearly overlap. In the case of FM coupling, some bands are localized at one defect, while the other set of bands are located at the other one.

In contrast, for the AFM coupling case, the same set of bands is located at the two defects. Thus, under an applied electric field the electronic behavior becomes identical for the FM and AFM couplings, regardless of the initial spin coupling. Furthermore, the magnetization for the FM case remains nearly constant with the field, while for the AFM case it tends to increase towards that of the FM case, as depicted in Fig. 4b. It is worth emphasizing that when employing gates, the spins of the defect lines in the array can readily be aligned, as they become parallel to each other, similar to the situation employing magnetic fields.

### Spin-polarization of valley states induced by n-doping

We now consider the effects of n-doping, including also the impact of electric fields. We explore the band structures under various n-doping values ranging from 0.3e to 1.3e. Figure 5 provides the bands represented for selected doping values. The previously degenerate bands, such as those corresponding to the defect and bilayer ones studied above, are now spin split, with a splitting related to the doping concentration. The defect bands remain at the Fermi level, but the splitting between bands with opposing spins increases; the spin-down bands shift upwards and the spin-up bands bend downwards, becoming partly occupied at the $\Gamma$ point. Since the BLG valley is displaced to lower energies, the spin-crossing point between the defect bands becomes occupied for n-doping values greater than 0.6e, and is therefore partly saturated, as one of the bands becomes almost flat. We find that the valley bands shift downwards in energy, so that the defect bands remain pinned at their energies. Furthermore, the downward shift of the valleys causes the associated valley and topological states to become spin-polarized.

We then assess the effect of electric field and n-doping on the defect line array. Figure 5 shows the resulting band structures for two different n-doping values as we vary from 0.3 to 1.30e with a high electric field of 150 mV/Å. The band structure is found to be nearly saturated within this regime, with a substantial energy gap. The defect bands are split, with the middle pair of spin-up and spin-down channels being almost degenerate. Doping causes a shift downwards in the energy of the valley bands, thereby lifting the spin-degeneracy of all bands.

#### Fermi nesting and charge modulation

**Figure 6:**
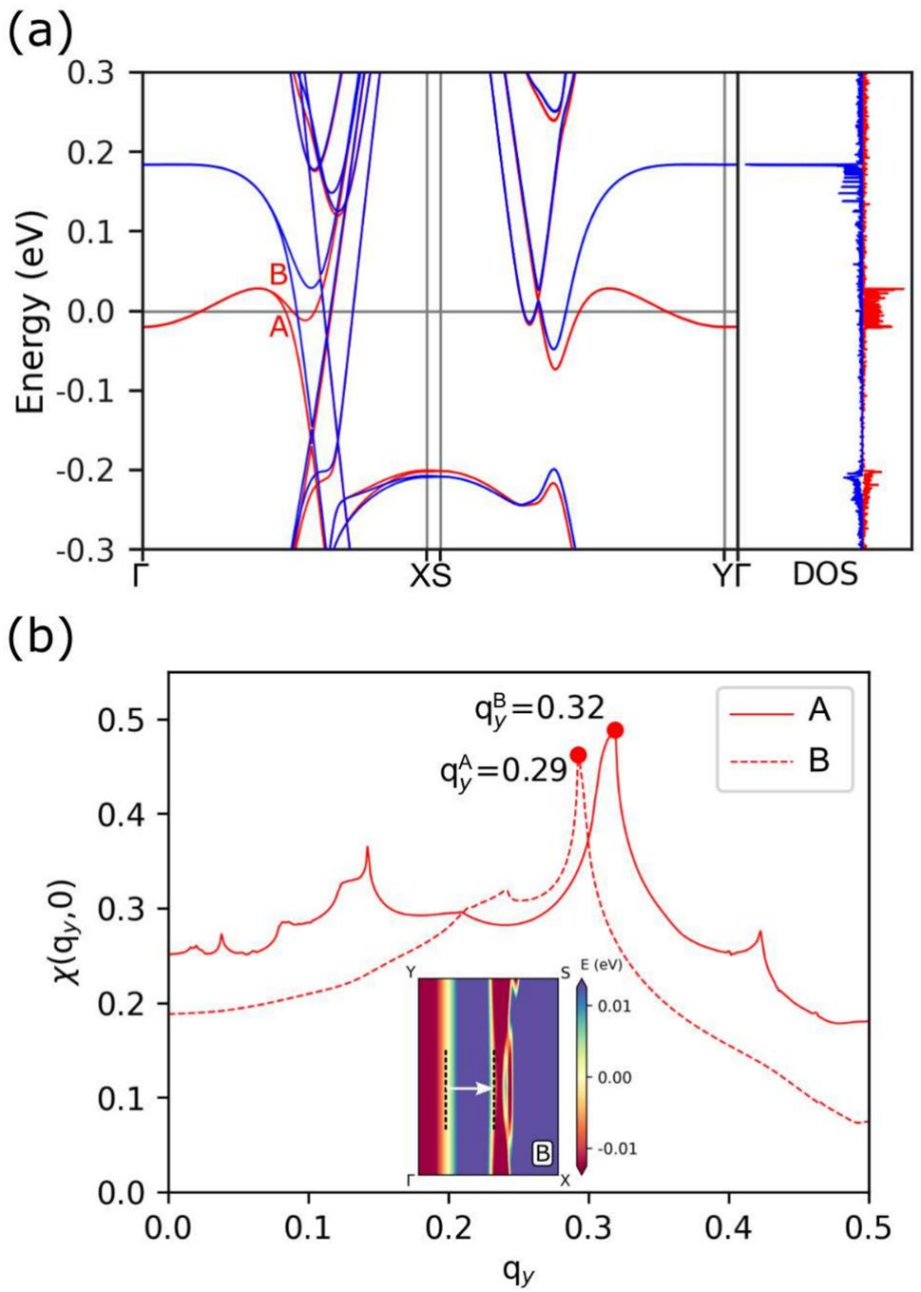
(**a**) Full band structure of the superlattice with n-doping of 1.0e along the 2D IBZ path. Red and blue colors denote the spin-up and spin-down bands, respectively. The right panel gives the spin-resolved density-of-states. Note that the spin-up defect bands are relevant near the Fermi level. The labels A and B indicate the two spin-up bands located in the vicinity of the Fermi energy. (**b**) Static response function of the spin-up bands A and B. The inset plots the B band in the 2D reciprocal zone around the Fermi energy; the arrow indicates the main nesting around $q_y \sim 0.33$. The peak in the B band indicates charge oscillations that can be commensurate with the defect line.

As previously discussed, n-doping induces both the downward shift and occupancy of spin-up defect bands, as well as partial occupancy of the valley states. We thus analyse the electron response function using the Lindhard theory. Figure 6a illustrates the band structure throughout high symmetry points in the full two-dimensional (2D) irreducible Brillouin zone (IBZ) for an n-doping value of 1.0e. We observe that the spin-up bands intersect the Fermi level multiple times along the path. The existence of electron and hole pockets on the Fermi surface suggests a probable nesting effect. The spin-resolved density of states shown in the right panel of Fig. 6a indeed confirms the key role of the spin-up bands near the Fermi level. The high density of states arises mainly from the two intertwined spin-up defect bands, identified as *A* and *B*, adjacent to the valleys and topological states near the K point.

We study the Fermi nestings through computations of the static response function $\chi (q)$, with q being the wave vector known as the momentum transfer associated with an electric field. The unit cell of the superlattice has a significant mismatch between the periodicity along the *x* and *y* directions. The SI contains 2D Fermi surface plots, with the case of the B band reproduced as an inset in Fig. 6b. These plots confirm that the quasi-1D nature of these states is evident, with parallel lines along $k_x$ that induce the maxima in the static response function. Figure 6b displays the response function $\chi (q_y)$ computed for the spin-up bands $\text {A}$ and $\text {B}$. It reveals significant peaks at $q^{\text {A}}_y=0.29$ and $q^{\text {B}}_y=0.32$ for the $\text {A}$ and $\text {B}$ bands, respectively.

Large values of $\chi (q_y)$ point to the possibility of charge density waves (CDW). Of particular interest is the situation when the CDW is commensurate with the lattice, meaning that the wavelength of the CDW is related to an integer number of unit lattice constants. We establish that $\lambda ^{CDW}_0=\frac{2\pi }{q_y}=\frac{N}{M}a_0$, where $a_0$ is the unit cell lattice, and *N* and *M* can be found as integers. In this case, a Peierls instability occurs, leading to distortions that can induce further transitions from the ground state to other states, especially when lower symmetries are considered. This is the case when larger cells along the line defects can be computed^37^. The wavelength $\lambda$ associated with $q^{A }_y$ is calculated as $\frac{N}{M}=4.36$ which is far from an integer value. This wavelength can be attributed to the interaction of the defect line with the topological state, and provides an opportunity to understand better the interaction of defects through topological states in general.

The $q^{B}_y$ value, however, corresponds to an $\frac{N}{M}=3.95\approx 4$, and is almost an integer. This is linked with the electron-electron interaction between the defect line and the valley states. On the one hand, going beyond these theoretical findings may lead to a Peierls instability, which may lead to a ground state with a distorted lattice, by allowing for a lower periodicity in charge densities using larger supercells. This may imply roughly four times the *y* lattice employed in this study, which is a large supercell beyond our calculations. On the other hand, scanning tunneling microscopy is currently a common method for investigating charge density oscillations in carbon nanostructures along defect lines^38,39^. This technique is similar to the approach we use to study confined states in quantum dots built of carbon nanotubes^40^. Notably, the two wavelengths may exhibit an interference pattern with beatings, which could be detected in measurements of commensurate and incommensurate regions along the line defects.

## Conclusions

In this work we study a periodic array of defect lines in bilayer graphene that lead to stacking changes, forming a superlattice with alternating AB and BA stacking domains. Our investigation of the superlattice electronic structure reveals the complex interplay between topological and defect line bands, which leads to some intriguing physical phenomena with electron-electron interaction playing a key role. The defect bands exhibit spin-momentum locking along the defect lines. Under an electric field, even if the spins of the defect bands have an initial antiferromagnetic coupling, they also tend to follow the trends observed in the ferromagnetic case. We then apply n-doping to the array, that leads to a downshift in energy of the bilayer valleys. This shift results in a partial occupation of the spin-up defect bands causing them to be pinned at the Fermi level. The states with spin momentum locking become occupied if the n-doping values exceed 1.0e, so the array of defect lines can play an interesting role as one-dimensional spin conducting channels. Additionally, the pinned bands become nested under n-doping, and we observe that they correspond to a nearly commensurate charge-density-wave, which could be potentially associated with Peierls instabilities. Since arrays of stacking grain boundaries such as 8-55 defect lines have gained considerable attention from the experimental community, we expect measurements in bilayers that confirm the charge and spin electron phenomena proposed herein.

## Supplementary Information

Supplementary Information.

## Data Availability

The data supporting this article have been included as part of the Supplementary Information.
