# Supplementary material for: Momentum-locked spin between topological and defect states in 1D patterns on bilayer graphene
Source: Sci Rep. 2025 Aug 1;15:28058. doi: 10.1038/s41598-025-12215-z (PMC12316863; doi:10.1038/s41598-025-12215-z)
Supplement: Supplementary file 1 — Supplementary Information. [file 41598_2025_12215_MOESM1_ESM.pdf]

Momentum-locked Spin Between Topological and Defect States in 1D Patterns on Bilayer Graphene

R. Guerrero-Avilés[1, 2], **A. Ayuela**[1,*], **Leonor Chico**[3], **W. Jaskólski**[4], **and M. Pelc**[4,*]

[1]Centro de Física de Materiales-Material Physics Center CFM-MPC, Donostia International Physics Center (DIPC), Paseo Manuel Lardizabal 4-5, 20018 Donostia-San Sebastián, Spain.
[2]TECNALIA, Basque Research and Technology Alliance (BRTA), 48160, Derio, Spain
[3]GISC, Departamento de Física de Materiales, Facultad de Ciencias Físicas, Universidad Complutense de Madrid, 28040 Madrid, Spain.
[4]Institute of Physics, Nicolaus Copernicus University in Toruń, Grudziadzka 5, 87-100 Toruń, Poland.
[*]Corresponding authors: a.ayuela@csic.es, mpelc@umk.pl

ABSTRACT

Structural Optimization

We perform structural relaxations to optimize geometries similar to the supercell presented in the Results section in the main text. Within the framework of density functional theory (DFT), we use the SIESTA method in conjunction with a vdw-DF2 functional to describe the non-local dispersion energies[1]. The geometry relaxations are performed using the following parameters: an energy cut-off of 600 Ry, a smearing of 10 meV, a $31\times31\times1$ k-mesh, and an ion forces tolerance of 1.9 meV/Å. As a result ,the interlayer distance is found to be 3.66 Åin agreement with previous results[2,3], and the geometry relaxations slightly increase almost all the intralayer carbon-carbon bond lengths. All these lengths are presented in a histogram in Fig. S1. We find that on average the carbon-carbon distances are slightly greater than that of pristine graphene (1.42 Å, which is shown as a dashed gray line).

In the lower panel, we focus on the lattice structure near the defect-line using colors to indicate the values in bond lengths. The large bond lengths are adjacent to the octagonal defects, while the small ones are in the pentagons. The bonds in the layer beneath the defect line are analogous to those farther away from the defect line in the top layer following symmetry considerations.

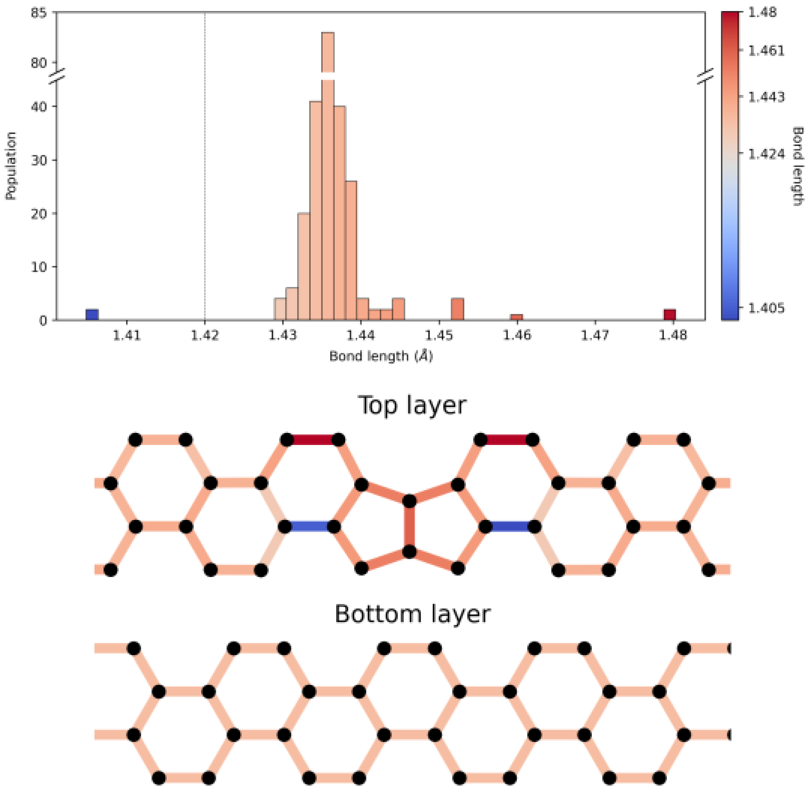

Figure S1. Bond lengths histogram for the relaxed defect line array geometry. The relaxed structure surrounding the defect line is displayed below with colors indicating bond lengths according to the histogram color bar.

Tight-Binding and Non-Polarized DFT Electronic Structure

We analyse the origin and response of the electron bands using electronic structure calculations performed on monolayer and bilayer systems. As a reference point, we additionally perform calculations on a monolayer containing defect lines. We use atomistic calculations employing both tight-binding (TB) and non-polarised DFT.

Regarding the TB method, we consider an intralayer hopping factor of $\gamma_0 = -2.66$ eV and an interlayer hopping factor of $\gamma_1 = 0.1\gamma_0$, following Ref. 4. The hopping factors are taken up to the first nearest neighbors both in the intralayer and interlayer cases. In addition, we use supercells to do both TB and DFT calculations. It is noteworthy that even in the monolayer scenario, we are dealing with an array of defect lines.

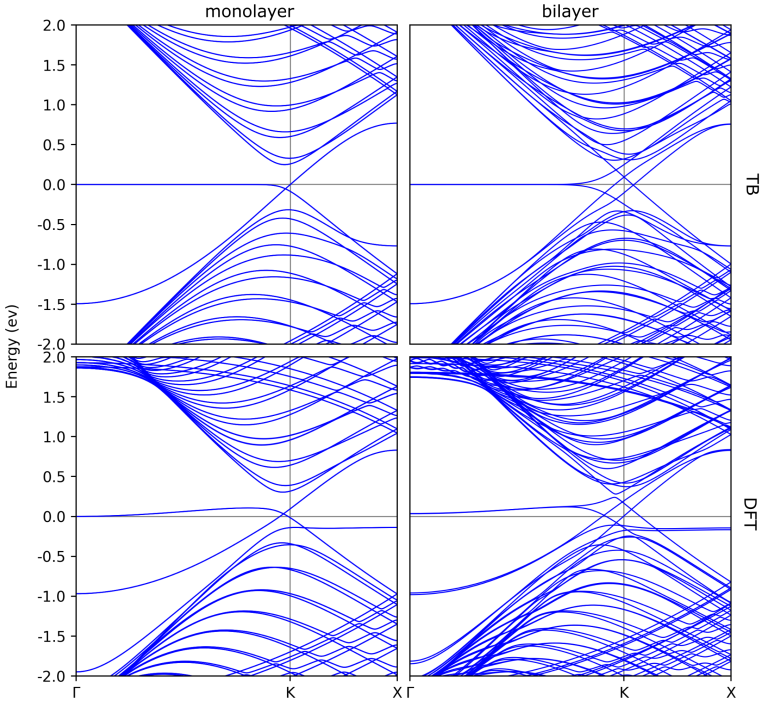

Figure S2. Electronic structure of an array of defect lines, with a length of $L = 24$ unit cells. Comparison between monolayer and bilayer cases calculated with tight-binding and non-polarised density functional theory.

We present our results in Fig. S2. We find that the bilayer bands with curvature at the K point have a gap where the defect states are lying, as shown in each panel. These defect states have the same origin as discussed in the Results section of the main text, and are related to zigzag nodes - at zero energy - and Klein nodes located in the energy range of -1.0 to -1.5 eV at the Γ point. Based on tight-binding calculations, the defect states in the monolayer case move below the Fermi level as the K point is approached. Around the same point, we have the main differences with the bilayer case that features four bands crossing at the gap.

A similar band behavior is observed in DFT and TB calculations. In the case of a monolayer, the defect band around the Fermi level is not fully flat due to the inclusion of electron-electron interactions in DFT. These interactions cause the crossing bands around the K point to move above the Fermi level for the monolayer. Similar trends are found for bilayers but, now with the bilayer topological states also coming into play. That is why there are further physical effects that deserve to be investigated by including electron-electron interaction beyond the tight-binding approach.

Fermi Surface Defect-Line States

We calculate the Lindhard function employing the Fermi surfaces obtained from DFT calculations. Here we focus on the Fermi surfaces of the occupied spin-up defect bands labeled A and B in the main text, subjected to n-doping (1.0e). To perform a

correct analysis of the Fermi surface, a better Brillouin sampling is required. Therefore, we set a fine k-mesh of $101\times15\times$ points using the Monkhorst-Pack procedure[5]. Since the A and B bands are next to the Fermi level, we use a smaller smearing broadening factor equal to 0.1 meV for these calculations.

Figure S3 shows the energy colormap of the A and B bands in the rectangular Brillouin zone. The lighter regions correspond to the Fermi surfaces exhibiting a quasi-1D-like shape. The Fermi surface edges that yield the largest contribution in the response function of the A and B bands are connected by arrows from left to right.

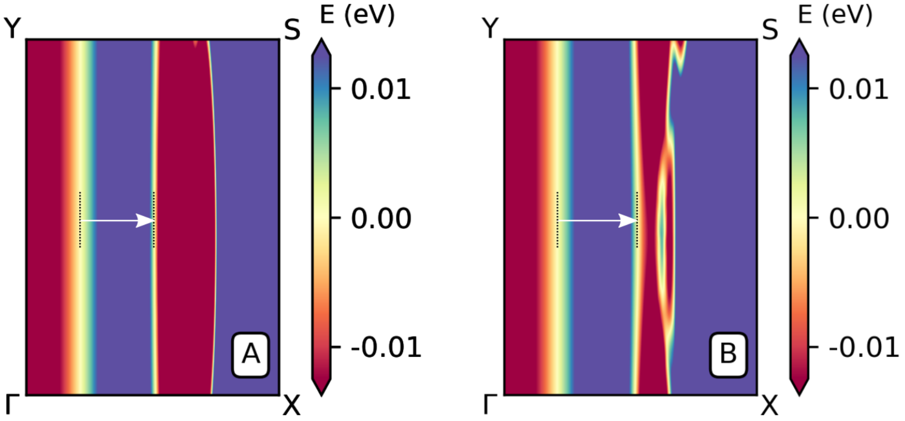

Figure S3. Fermi surface in the entire rectangular Brillouin zone of the *A* and *B* defect bands displayed in Fig. 6 of the main text.

References

1. Lee, K., Murray, E. D., Kong, L., Lundqvist, B. I. & Langreth, D. C. Higher-accuracy Van der Waals density functional. *Phys. Rev. B* **82**, 081101 (2010).
2. Geisenhof, F. R. *et al.* Anisotropic Strain-Induced Soliton Movement Changes Stacking Order and Band Structure of Graphene Multilayers: Implications for Charge Transport. *ACS Appl. Nano Mater.* **2**, 6067–6075, DOI: 10.1021/acsanm.9b01603 (2019). Publisher: American Chemical Society.
3. Guerrero-Avilés, R., Pelc, M., Geisenhof, F. R., Weitz, R. T. & Ayuela, A. Rhombohedral trilayer graphene is more stable than its Bernal counterpart. *Nanoscale* **14**, 16295–16302, DOI: 10.1039/D2NR01985J (2022). Publisher: The Royal Society of Chemistry.
4. Jaskólski, W., Pelc, M., Chico, L. & Ayuela, A. Existence of nontrivial topologically protected states at grain boundaries in bilayer graphene: signatures and electrical switching. *Nanoscale* **8**, 6079 (2016).
5. Monkhorst, H. J. & Pack, J. D. Special points for Brillouin-zone integrations. *Phys. Rev. B* **13**, 5188 (1976).
